# Supplementary material for: Intention to Vaccinate against COVID-19 in Adolescents: A Systematic Review
Source: Vaccines (Basel). 2023 Aug 21;11(8):1393. doi: 10.3390/vaccines11081393 (PMC10458082; doi:10.3390/vaccines11081393)
Supplement: Supplementary file 1 [file vaccines-11-01393-s001.zip › vaccines-2518955-supplementary.pdf]

Supplementary Materials

**Features**	MeSH Terms	Synonyms
COVID	COVID-19	2019 Novel Coronavirus Disease<br>2019 Novel Coronavirus Infection<br>2019-nCoV Disease<br>2019-nCoV Infection<br>COVID-19 Pandemic<br>COVID-19 Pandemics<br>COVID-19 Virus Disease<br>COVID-19 Virus Infection<br>COVID19<br>Coronavirus Disease 2019<br>Coronavirus Disease-19<br>SARS Coronavirus 2 Infection<br>SARS-CoV-2 Infection
Vaccination/ Vaccine	Vaccination<br>Vaccines	Immunization, Active<br>Vaccine
Adolescent	Adolescent	Adolescence<br>Adolescents<br>Adolescents, Female<br>Adolescents, Male<br>Teenagers<br>Teens<br>Youth

Search Strategy (PubMed)

1 "covid 19"[MeSH Terms]

2 "covid 19"[MeSH Terms] OR "covid 19"[All Fields] OR "2019 novel coronavirus disease"[All Fields] OR "covid 19"[MeSH Terms] OR "covid 19"[All Fields] OR "2019 novel coronavirus infection"[All Fields] OR "covid 19"[MeSH Terms] OR "covid 19"[All Fields] OR "2019 ncov disease"[All Fields] OR "covid 19"[MeSH Terms] OR "covid 19"[All Fields] OR "2019 ncov infection"[All Fields] OR "covid 19"[MeSH Terms] OR "covid 19"[All Fields] OR "covid 19 pandemic"[All Fields] OR "covid 19"[MeSH Terms] OR "covid 19"[All Fields] OR "covid 19 pandemics"[All Fields] OR "covid 19"[MeSH Terms] OR "covid 19"[All Fields] OR "covid 19 virus disease"[All Fields] OR "covid 19"[MeSH Terms] OR "covid 19"[All Fields] OR "covid 19 virus infection"[All Fields] OR "covid 19"[MeSH Terms] OR "covid 19"[All Fields] OR "covid19"[All Fields] OR "covid 19"[MeSH Terms] OR "covid 19"[All Fields] OR "coronavirus disease 2019"[All Fields] OR "covid 19"[MeSH Terms] OR "covid 19"[All Fields] OR "coronavirus disease 19"[All Fields] OR "covid 19"[MeSH Terms] OR "covid 19"[All Fields] OR "sars coronavirus 2 infection"[All Fields] OR "covid 19"[MeSH Terms] OR "covid 19"[All Fields] OR "sars cov 2 infection"[All Fields]

3 1 or 2

4 "vaccination"[MeSH Terms] OR "vaccines"[MeSH Terms]

5 "vaccination"[MeSH Terms] OR "vaccination"[All Fields] OR ("immunization"[All Fields] AND "active"[All Fields]) OR "immunization active"[All Fields] OR ("vaccin"[Supplementary Concept] OR "vaccin"[All Fields] OR "vaccination"[MeSH Terms] OR "vaccination"[All Fields] OR "vaccinable"[All Fields] OR "vaccinal"[All Fields] OR "vaccinate"[All Fields] OR "vaccinated"[All Fields] OR "vaccinates"[All Fields] OR "vaccinating"[All Fields] OR "vaccinations"[All Fields] OR "vaccination s"[All Fields] OR "vaccinator"[All Fields] OR "vaccinators"[All Fields] OR "vaccine s"[All Fields] OR "vaccined"[All Fields] OR "vaccines"[MeSH Terms] OR "vaccines"[All Fields] OR "vaccine"[All Fields] OR "vaccins"[All Fields])

6 4 or 5

7 "adolescent"[MeSH Terms]

8 "adolescences"[All Fields] OR "adolescency"[All Fields] OR "adolescent"[MeSH Terms] OR "adolescent"[All Fields] OR "adolescence"[All Fields] OR "adolescents"[All Fields] OR "adolescent s"[All Fields] OR ("adolescences"[All Fields] OR "adolescency"[All Fields] OR "adolescent"[MeSH Terms] OR "adolescent"[All Fields] OR "adolescence"[All Fields] OR "adolescents"[All Fields] OR "adolescent s"[All Fields]) OR ("adolescent"[MeSH Terms] OR "adolescent"[All Fields] OR ("adolescents"[All Fields] AND "female"[All Fields]) OR "adolescents female"[All Fields]) OR ("adolescent"[MeSH Terms] OR "adolescent"[All Fields] OR ("adolescents"[All Fields] AND "male"[All Fields]) OR "adolescents male"[All Fields]) OR ("adolescent"[MeSH Terms] OR "adolescent"[All Fields] OR "teenage"[All Fields] OR "teenager"[All Fields] OR "teenagers"[All Fields] OR "teenaged"[All Fields] OR "teenager s"[All Fields] OR "teenages"[All Fields]) OR ("adolescent"[MeSH Terms] OR "adolescent"[All Fields] OR "teens"[All Fields] OR "teen s"[All Fields]) OR ("adolescent"[MeSH Terms] OR "adolescent"[All Fields] OR "youth"[All Fields] OR "youths"[All Fields] OR "youth s"[All Fields])

9 7 or 8

10 "willingness"[All Fields] OR "accept"[All Fields] OR "acceptabilities"[All Fields] OR "acceptability"[All Fields] OR "acceptable"[All Fields] OR "acceptably"[All Fields] OR "acceptance"[All Fields] OR "acceptances"[All Fields] OR "acceptation"[All Fields] OR

"accepted"[All Fields] OR "accepter"[All Fields] OR "accepters"[All Fields] OR "accepting"[All Fields] OR "accepts"[All Fields] OR "accept"[All Fields] OR "acceptabilities"[All Fields] OR "acceptability"[All Fields] OR "acceptable"[All Fields] OR "acceptably"[All Fields] OR "acceptance"[All Fields] OR "acceptances"[All Fields] OR "acceptation"[All Fields] OR "accepted"[All Fields] OR "accepter"[All Fields] OR "accepters"[All Fields] OR "accepting"[All Fields] OR "accepts"[All Fields] OR "hesitance"[All Fields] OR "hesitancies"[All Fields] OR "hesitancy"[All Fields] OR "hesitant"[All Fields] OR "hesitate"[All Fields] OR "hesitated"[All Fields] OR "hesitating"[All Fields] OR "hesitation"[All Fields] OR "hesitations"[All Fields] OR "barrier"[All Fields] OR "barrier s"[All Fields] OR "barriers"[All Fields] OR "enable"[All Fields] OR "enabled"[All Fields] OR "enablement"[All Fields] OR "enablements"[All Fields] OR "enabler"[All Fields] OR "enablers"[All Fields] OR "enables"[All Fields] OR "enabling"[All Fields] OR "attitude"[MeSH Terms] OR "attitude"[All Fields] OR "attitudes"[All Fields] OR "attitude s"[All Fields] OR "percept"[All Fields] OR "perceptibility"[All Fields] OR "perceptible"[All Fields] OR "perception"[MeSH Terms] OR "perception"[All Fields] OR "perceptions"[All Fields] OR "perceptional"[All Fields] OR "perceptive"[All Fields] OR "perceptiveness"[All Fields] OR "percepts"[All Fields]
11 3 and 6 and 9 and 10
12 Filter: English

[594 results]

Search Strategy (Medline)

1. exp COVID-19/
2. (2019 Novel Coronavirus Disease or 2019 Novel Coronavirus Infection or 2019-nCoV Disease or 2019-nCoV Infection or COVID-19 Pandemic or COVID-19 Pandemics or COVID-19 Virus Disease or COVID-19 Virus Infection or COVID19 or Coronavirus Disease 2019 or Coronavirus Disease-19 or SARS Coronavirus 2 Infection or SARS-CoV-2 Infection).mp. [mp=title, abstract, heading word, drug trade name, original title, device manufacturer, drug manufacturer, device trade name, keyword, floating subheading word, candidate term word]
3. 1 or 2
4. exp Vaccination/
5. exp Vaccines/
6. (Immunization, Active or Vaccine).mp. [mp=title, abstract, heading word, drug trade name, original title, device manufacturer, drug manufacturer, device trade name, keyword, floating subheading word, candidate term word]
7. 4 or 5 or 6
8. exp Adolescent/
9. exp Young Adult/
10. (Adolescence or Adolescents or Adolescents, Female or Adolescents, Male or Teenagers or Teens or Youth).mp. [mp=title, abstract, heading word, drug trade name, original title, device manufacturer, drug manufacturer, device trade name, keyword, floating subheading word, candidate term word]
11. 8 or 9 or 10
12. (willingness or acceptance or acceptability or hesitancy or barriers or enablers or perceptions or attitudes).mp. [mp=title, abstract, heading word, drug trade name, original title, device manufacturer, drug manufacturer, device trade name, keyword, floating subheading word, candidate term word]
13. 3 and 7 and 11 and 12
14. limit 13 to english language

[574 results]

Search Strategy (EMBASE)

1. exp coronavirus disease 2019/
2. (2019 Novel Coronavirus Disease or 2019 Novel Coronavirus Infection or 2019-nCoV Disease or 2019-nCoV Infection or COVID-19 Pandemic or COVID-19 Pandemics or COVID-19 Virus Disease or COVID-19 Virus Infection or COVID19 or Coronavirus Disease 2019 or Coronavirus Disease-19 or SARS Coronavirus 2 Infection or SARS-CoV-2 Infection).mp. [mp=title, abstract, heading word, drug trade name, original title, device manufacturer, drug manufacturer, device trade name, keyword heading word, floating subheading word, candidate term word]
3. 1 or 2
4. exp vaccination/
5. exp vaccine/
6. (Immunization, Active or Vaccine).mp. [mp=title, abstract, heading word, drug trade name, original title, device manufacturer, drug manufacturer, device trade name, keyword heading word, floating subheading word, candidate term word]
7. 4 or 5 or 6
8. exp adolescent/
9. exp young adult/
10. (Adolescence or Adolescents or Adolescents, Female or Adolescents, Male or Teenagers or Teens or Youth).mp. [mp=title, abstract, heading word, drug trade name, original title, device manufacturer, drug manufacturer, device trade name, keyword heading word, floating subheading word, candidate term word]
11. 8 or 9 or 10
12. (willingness or acceptance or acceptability or hesitancy or barriers or enablers or perceptions or attitudes).mp. [mp=title, abstract, heading word, drug trade name, original title, device manufacturer, drug manufacturer, device trade name, keyword heading word, floating subheading word, candidate term word]
13. 3 and 7 and 11 and 12

[537 results]

Search Strategy (CINAHL)

S1 MH COVID-19 OR (2019 Novel Coronavirus Disease or 2019 Novel Coronavirus Infection or 2019-nCoV Disease or 2019-nCoV Infection or COVID-19 Pandemic or COVID-19 Pandemics or COVID-19 Virus Disease or COVID-19 Virus Infection or COVID19 or Coronavirus Disease 2019 or Coronavirus Disease-19 or SARS Coronavirus 2 Infection or SARS-CoV-2 Infection)

S2 MH Vaccination OR MH Vaccines OR (Immunization, Active or Vaccine)

S3 MH Adolescent OR MH Young adult OR (Adolescence or Adolescents or Adolescents, Female or Adolescents, Male or Teenagers or Teens or Youth)

S4 willingness or acceptance or acceptability or hesitancy or barriers or enablers or perceptions or attitudes

S5 (willingness or acceptance or acceptability or hesitancy or barriers or enablers or perceptions or attitudes) AND (S1 AND S2 AND S3 AND S4)

[58 results]
